# Evaluation of Composites Comprising Spherical, Porous, Sintered β-Tricalcium Phosphate Particles and Cyanoacrylate as Bone Cement

**DOI:** 10.3390/jfb16120458

**Published:** 2025-12-09

**Authors:** Kazuaki Hashimoto, Shuhei Aida, Iori Takigawa, Hirobumi Shibata, Satoshi Kobune, Toshiisa Konishi, Takashi Meguro, Shigeo Fukuyama, Shinya Tanaka

**Affiliations:** 1 Department of Applied Chemistry, Faculty of Engineering, Chiba Institute of Technology, 2-17-1 Tsudanuma, Narashino 275-0016, Chiba, Japan; s.aida@aion-kk.co.jp (S.A.); takigawaiori0824@gmail.com (I.T.); hirobumi.shibata@p.chibakoudai.jp (H.S.); 2 Tokyo Metropolitan Industrial Technology Research Institute, 2-4-10, Aomi, Koto-ku 135-0064, Tokyo, Japan; kobune.satoshi@iri-tokyo.jp (S.K.); konishi.toshiisa@iri-tokyo.jp (T.K.); 3 Fukuyama Medical Co., Ltd., 1-11-1, Chishirodai-nishi, Wakaba-ku, Chiba 274-0004, Chiba, Japan; meguro@fukuyamaika.com (T.M.); s.fukuyama@fukuyamaika.com (S.F.); 4 Department for Orthopedic Surgery and Center for Joint Replacement, Toto Kasukabe Hospital, 652-7, Ohata, Kasukabe 344-0022, Saitama, Japan; uf8s-tnk@asahi-net.or.jp

**Keywords:** β-tricalcium phosphate, cyanoacrylate adhesive, injectable bone cement, porous spherical sintered granules, curing behavior, mechanical properties

## Abstract

Bone cements based on polymethyl methacrylate (PMMA) remain the clinical standard for joint replacement and vertebral augmentation but suffer from several major challenges. These include excessive stiffness compared with cancellous bone, lack of resorption and osteoconductivity, and thermal necrosis during curing. Calcium phosphate cements (CPCs) are bioactive and resorbable but tend to exhibit low mechanical strength, poor injectability and brittle fracture. The work reported herein developed an injectable composite bone cement by combining spherical, porous, sintered β-tricalcium phosphate (β-TCP) particles with a cyanoacrylate adhesive. The β-TCP granules provided bioactivity and a favorable microarchitecture while the cyanoacrylate ensured strong adhesion and rapid setting. Ion substitution with Mg, Na and Si was found to modify the surface acidity of the material while also inhibiting cyanoacrylate polymerization, thereby extending the setting time and lowering the exotherm temperature. This composite exhibited high chemical stability, smooth injectability and early surface reactivity indicative of osteoconductivity. The compressive strength of the material stabilized at approximately 40 MPa and so exceeded that of cancellous bone. This new material also showed ductility, energy absorption and superior impact resistance, although its tensile and fatigue resistance remained limited. Importantly, the composite provided strength comparable to that of PMMA in cemented models during fixation tests and significantly outperformed CPCs in cementless tibial tray fixation experiments. These findings demonstrate that the present β-TCP/cyanoacrylate cement bridges the gap between PMMA and CPCs by combining injectability and mechanical reliability with bioactivity. This cement is therefore a promising next-generation option for minimally invasive osteoporotic fracture treatment and revision arthroplasty.

## 1. Introduction

Artificial joint replacement (AJR) is a well-established and effective therapeutic intervention for degenerative joint diseases such as osteoarthritis, rheumatoid arthritis and avascular necrosis of the femoral head, offering substantial improvements in pain relief and functional recovery [1]. Even so, the long-term outcomes of AJR often involve challenges related to complications that necessitate revision surgery. The major causes of revision surgery can be classified into five categories: (1) implant loosening due to aseptic osteolysis triggered by wear particles; (2) periprosthetic joint infection (PJI) resulting from bacterial colonization and biofilm formation; (3) implant dislocation or instability caused by malposition or soft tissue imbalance; (4) mechanical failure of implant components due to wear or breakage; and (5) periprosthetic fractures, particularly in elderly patients with compromised bone quality [2,3,4,5,6]. Revision surgery presents greater technical complexity than primary arthroplasty, as the former often involves managing extensive bone loss, infection or implant instability. Polymethyl methacrylate-based bone cement (PMMA-based BC) remains a fundamental component of fixation strategies, especially in cases involving poor bone quality or defects. Recent reviews and textbooks have examined the principles, techniques and outcomes of cemented arthroplasty [7,8,9,10]. Depending on the clinical scenario, revision procedures may require complete removal of the existing cement or re-cementation using the cement-in-cement technique. While the former offers improved bonding at the bone–cement interface, it is technically demanding. The latter allows for reduced operative time and minimized bone damage but may compromise fixation strength [11]. Cemented long stems are frequently used for femoral fixation during revision surgeries, regardless of whether the primary surgery was cemented or uncemented [12]. In the case of infection, antibiotic-loaded bone cement (ALBC), typically containing aminoglycosides or vancomycin, is employed to achieve local antimicrobial activity [13,14]. The increasing global prevalence of osteoporosis represents a major public health concern, particularly in aging populations. Osteoporosis is characterized by reduced bone mass and deterioration of the bone microarchitecture, resulting in decreased bone strength and an elevated risk of fracture [15]. An estimated 200 million individuals are affected worldwide, most of whom are postmenopausal women [16]. This trend underscores the urgent need for effective strategies to prevent and treat osteoporotic fractures, which have profound implications not only for individual quality of life but also for healthcare systems burdened by associated costs [17]. Among the various osteoporotic fractures, osteoporotic vertebral compression fractures (OVCFs) are especially prevalent and debilitating. OVCFs occur when weakened vertebral bodies collapse under physiological loading or minor trauma, leading to pain, spinal deformity and reduced mobility [18,19]. Although conservative treatments such as analgesics and spinal orthoses have traditionally been used, these approaches often fail to provide sufficient pain relief or prevent further fractures [20]. Consequently, minimally invasive procedures such as percutaneous vertebroplasty (PVP) and balloon kyphoplasty (BKP) have been widely adopted. These techniques involve the injection of bone cement into the affected vertebra to restore mechanical stability and facilitate early mobilization [21]. PMMA-based BC exhibits rapid polymerization, excellent moldability and established clinical performance, and so is the most widely used material in both PVP and BKP procedures [22]. However, several intrinsic limitations of PMMA-based BC compromise the long-term safety and effectiveness of this material. Firstly, the elastic modulus of PMMA (1700–3700 MPa) [23] is significantly higher than that of cancellous bone (10–900 MPa) [24], resulting in stress shielding and an increased risk of adjacent vertebral fractures. Secondly, PMMA is a non-resorbable material and so its presence can lead to interfacial gaps, fibrous encapsulation and inflammatory reactions over time [25]. Thirdly, the exothermic polymerization of this compound poses a risk of thermal injury to surrounding bone and neural tissue. Moreover, PMMA lacks osteoconductivity and biocompatibility, thereby limiting its capacity to promote new bone formation [26]. In response to these challenges, calcium phosphate cements (CPCs) have been developed as bioactive, resorbable alternatives to PMMA-based BC. CPCs, typically based on hydroxyapatite (HAp), brushite (dicalcium phosphate dihydrate, DCPD) or α/β-tricalcium phosphate (TCP), set via dissolution–precipitation reactions in aqueous environments to form a bone-like apatite phase [27,28]. These materials show exceptional biocompatibility and osteoconductivity and can also be resorbed and replaced by newly formed bone, all of which are highly desirable features promoting long-term integration [29]. Nevertheless, the relatively low mechanical strength, poor injectability and brittleness of these compounds limit their widespread use [30,31,32]. Recent review papers have emphasized the continuous development of injectable CPCs and bioactive composite cements, underscoring advances in polymer reinforcement, ion substitution and hybrid designs aimed at addressing problems related to brittleness and limited injectability [33,34]. At the same time, new biomaterial-based strategies have emerged that focus on modulating the bone microenvironment and enhancing biological responses in addition to mechanical fixation [35]. Based on these trends, the present study demonstrates a distinct approach that bridges the use of adhesive-based fixation and bioactive ceramics.

The present work demonstrates a novel composite BC comprising spherical, porous, sintered particles of β-tricalcium phosphate (β-TCP) combined with a cyanoacrylate adhesive. β-TCP is a resorbable calcium phosphate ceramic that provides a high degree of biocompatibility together with good osteoconductivity and is already widely used in clinical settings for bone regeneration. The use of spherical, porous β-TCP particles was expected to enhance cell infiltration and promote new bone formation by providing a favorable microarchitecture [36]. Cyanoacrylate is a medically approved tissue adhesive that offers rapid curing, high adhesive strength and suitable tissue compatibility. The integration of this adhesive with β-TCP was intended to yield a hybrid material combining the injectability and mechanical reliability of the synthetic adhesive with the bioactivity and resorption ability of the calcium phosphate ceramic.

Despite the extensive use of PMMA-based bone cements, calcium phosphate cements, and cyanoacrylate adhesives, no existing material simultaneously satisfies the clinical requirements of injectability, controlled curing behavior, bioactivity, and mechanical reliability. To date, cyanoacrylate-based cements have remained unsuitable for orthopedic applications because their anionic polymerization is initiated too rapidly by ionic species on ceramic surfaces, resulting in excessively short working times and high exothermic heat. Likewise, no previous study has demonstrated a strategy to modulate cyanoacrylate polymerization kinetics through rational control of β-TCP surface chemistry. The present work introduces a conceptually new approach: integrating a resorbable, osteoconductive β-TCP granular phase with a cyanoacrylate adhesive and regulating polymerization through Mg-, Na-, and Si-based ion substitution. To the best of our knowledge, this is the first study to show that cyanoacrylate polymerization can be systematically controlled by engineering the Brønsted/Lewis acidity and ionic environment of β-TCP particles, thereby enabling extended workable time, reduced exothermic temperature, and enhanced mechanical toughness in an injectable bone cement.

The aim of this study was to develop an injectable β-TCP/cyanoacrylate composite bone cement and to evaluate its polymerization behavior, microstructure, mechanical properties, and fixation performance.

## 2. Materials and Methods

### 2.1. Synthesis and Sintering of Materials

#### 2.1.1. Preparation of Raw Powder

The composition of the raw powder mixture is summarized in Table 1. β-Tricalcium phosphate (β-TCP) was synthesized by mixing calcium carbonate (CaCO_3_; FUJIFILM Wako Pure Chemical Corporation, Osaka, Japan) and ammonium hydrogen phosphate ((NH_4_)_2_HPO_4_; FUJIFILM Wako) at a Ca/P molar ratio of 1.50. The precursor mixtures were subjected to wet milling prior to calcination. Wet milling was conducted using a rotary ball mill (ANZ-100S, Nitto-kagaku Co., Ltd., Nagoya, Japan) equipped with an Al_2_O_3_ ceramic pot. Nylon balls (φ15 mm and φ20 mm, 1 kg each) were used as the grinding media. Ethanol (purity: 99.95%, FUJIFILM Wako) was added as the solvent. The milling was performed at a rotation speed of 66 rpm for 70 h. After the ethanol was removed using a rotary evaporator (N-1300, EYELA, Tokyo, Japan), the precursor was sintered at 1100 °C for 3 h, employing heating and cooling rates of 5 °C/min. Mg-β-TCP specimens were prepared by substituting magnesium oxide (MgO) into the precursor composition, whereas MgNa-β-TCP/Si specimens were synthesized by incorporating MgO, sodium nitrate (NaNO_3_) and silicon dioxide (SiO_2_). The amounts of these dopants were adjusted to yield a (Ca + Na + Mg + □)/(P + Si) ratio of 1.571, where □ denotes a cation vacancy. The resulting mixtures were dried and sintered at 1130 °C for 3 h. Mg-β-TCP and MgNa-β-TCP/Si exhibited higher thermal stability, because the substitutional incorporation of Mg^2+^ ions into the Ca(5) sites acts as a structural stabilizer [37]. Owing to this stabilization effect, these substituted samples do not undergo phase transformation to α-TCP even when sintered at 1130 °C, which allows full densification at this temperature. In contrast, pure β-TCP partially transforms into α-TCP when sintered at 1130 °C. Therefore, to avoid the β → α phase transition while still achieving sufficient sintering, we selected 1100 °C as the optimal temperature for pure β-TCP. The phases in the sintered powders were identified using X-ray diffraction (XRD, MiniFlex-600, Rigaku, Tokyo, Japan; Scan speed: 5°/min., Step width: 0.02°) while the functional groups in the specimens were analyzed using Fourier transform infrared spectroscopy (FT-IR, FT/IR-4X, JASCO, Tokyo, Japan; FT-IR resolution: 4 cm^−1^, Number of scans: 32). The sample microstructures were observed by scanning electron microscopy (SEM, JSM-7800, JEOL, Tokyo, Japan). The solid-state reaction equation for the raw material in this experiment is shown below.

(1) $$21\;CaCO_{3}+14\;(NH_{4}{)}_{2}HPO_{4}\to Ca_{21}□(PO_{4}{)}_{14}+21CO_{2}+28NH_{3}+21\;H_{2}O$$

(2) $$19\;CaCO_{3}+14\;(NH_{4}{)}_{2}HPO_{4}+2MgO\to Ca_{19}{Mg}_{2}□(PO_{4}{)}_{14}+19\;CO_{2}+28\;NH_{3}+21\;H_{2}O$$

(3) $$19\;CaCO_{3}+13.58\;(NH_{4}{)}_{2}HPO_{4}+2MgO+0.42Na\left(NO_{3}\right)+0.42SiO_{2}\;\to Ca_{19}{Mg}_{2}{Na}_{0.42}□0.58(PO_{4}{)}_{13.58}(SiO_{4}{)}_{0.42}+19\;CO_{2}+27.16\;NH_{3}+20.37\;H_{2}O\;+0.315O_{2}+0.42\;NO$$

#### 2.1.2. Preparation of Spherical Sintered Particles

Quantities of the sintered β-TCP, Mg_9.0_-β-TCP or MgNa-β-TCP/Si powders were pulverized in a mortar for 30 min. Each powder was then suspended in a 5 wt% polyvinyl alcohol (PVA; FUJIFILM Wako) solution and processed using a spray dryer (LT-8, Okawara Chemical Machinery, Yokohama, Japan). The spray-drying parameters comprised an inlet temperature of 250 °C, outlet temperature of 100 °C, atomizer speed of 20,000 rpm and feed rate of 50 mL/min (see Figure S1). The dried granules were subsequently sintered in rectangular alumina crucibles (120 × 40 mm) at 1100 °C (in the case of the β-TCP) or 1130 °C (in all other cases) for 48 h to obtain spherical, porous, sintered particles. The resulting structures were characterized by XRD (MiniFlex-600, Rigaku), Rietveld analysis (Rigaku PDXL 2 version 2.1.3.6 attached Rietveld software) and FT-IR (FT/IR-4X, JASCO) and SEM (JSM-7800, JEOL).

### 2.2. Setting Tests

Mixtures of an ethyl cyanoacrylate adhesive (Aron Alpha 241, Toa Gosei, Nagoya, Japan) and the spherical sintered particles of β-TCP, Mg_9.0_-β-TCP or MgNa-β-TCP/Si were prepared at liquid-to-powder (L/P) ratios ranging from 0.7 to 1.0 mL/g at 23 ± 5 °C and a relative humidity below 75%. Mixing was performed inside a 5 mL syringe (SS-05SZ, Terumo, Tokyo, Japan) using a disposable stirrer (ART.00939-00, Kartell SpA, Milan, Italy) for 2 min. Each mixture was subsequently injected into a silicone tube (inner diameter 8 mm, height 20 mm). The curing behavior of each specimen was assessed by probing the surface with a Gilmore needle (B-001, JAPAN MECC, Tokyo, Japan) at 1 min intervals. The setting time was defined as the point at which the needle no longer penetrated the surface. The exotherm for each reaction was monitored using an infrared thermometer (IR-TE2, Chino, Tokyo, Japan). After curing, specimens were machined into cylinders (diameter 8 mm, height 10 mm) with parallel end surfaces in preparation for testing. The cured BC samples were evaluated using SEM (JSM-7800, JEOL) and electron probe microanalysis (EPMA; JXA-8800, JEOL).

### 2.3. Semi-Quantification of Acid Sites on Sintered Particles

Brønsted and Lewis acid sites on the surfaces of the various sintered particles were evaluated using FT-IR spectroscopy following pyridine adsorption. In each trial, a 1 g quantity of particles was immersed in pyridine (FUJIFILM Wako) with stirring for 6 h at 23 ± 5 °C. Each sample was recovered by vacuum filtration, dried for 12 h and then analyzed. The intensities of the peaks at approximately 1500 cm^−1^ (related to pyridinium ions) and 1450 cm^−1^ (related to coordinated pyridine) in the resulting spectra were used to quantify Brønsted and Lewis acid sites based on comparisons to the C-H stretching peak at 2925 cm^−1^. The concentrations of surface acid groups were also assessed by dispersing 1 g of particles in 20 mL of ultrapure water with stirring for 10 min, following which the pH of the water was determined using a pH meter (F-54, Horiba, Tokyo, Japan).

### 2.4. Water Stability of BC Paste

Cured specimens were immersed in phosphate-buffered saline (PBS; FUJIFILM Wako) at 37 °C for 336 h. The masses before (W_0_) and after (W_a_) immersion were determined using an analytical balance having an accuracy of 0.1 mg while the percentage mass retained was calculated as (W_a_/W_0_) × 100.

### 2.5. Mechanical Properties of Cured BC Specimens

Cured BC specimens were prepared by mixing the adhesive and particles at an L/P ratio of 0.7 mL/g for 90 s and then pouring the mixtures into fluorine resin molds. The molds were held at a temperature of 23 ± 5 °C and a relative humidity of 75% or less for 24 h before demolding. After removal, each specimen was immersed in a phosphate-buffered solution at 23 ± 5 °C for 96 to 168 h. Following immersion, each sample was dried at 23 ± 5 °C and a relative humidity of 75% or less for 16 to 24 h. To ensure that the surface condition of each specimen did not affect the test results, the samples were polished by grinding prior to assessment.

Figure S2 shows the dimensions and shapes of each type of test specimen. Tensile and compression tests were conducted using a uniaxial testing machine (AG-100kNXplus, Shimadzu Corporation, Kyoto, Japan) under static loading conditions with initial strain rates of 1 × 10^−3^ to 3 × 10^−3^ s^−1^. The elastic modulus was calculated using data from strain gauges attached to the specimen surfaces. Bending tests were conducted using a three-point bending method with a support distance of 15 mm and a crosshead speed of 0.02 mm/s. Torsion tests were performed using a torsion testing machine (TTM-3000N mI, Shimadzu Corporation, Kyoto, Japan) in conjunction with dumbbell-shaped test specimens made by machining cylindrical cured materials. For comparison purposes, test pieces were also prepared from commercially available bone fillers. It should be noted that tensile and fatigue test pieces made from this material were excluded from the comparative evaluation because these items were difficult to remove from the fluoroplastic molds. In addition, it was difficult to turn the torsion test pieces after molding with fluoroplastic, and so specimens made from the commercially available product could not be tested. A custom-made water bath was installed in a uniaxial testing machine (E10000, Instron, Norwood, MA, USA) to simulate a biological environment for fatigue testing, during which stress amplitude was applied with each specimen immersed in a phosphate buffer solution at 37 °C. For comparison purposes, evaluations were also conducted in air. The fatigue test conditions consisted of a stress ratio of *R* = −1 and a frequency of 5 to 10 Hz. A calibrated Charpy impact testing machine (MC-0.5P-1, Maekawa Testing Machine Mfg, Co., Ltd., Tokyo, Japan) was used to acquire fracture toughness data. This apparatus was equipped with a load sensor on the hammer, enabling evaluation of the impact force at the moment at which the hammer struck the test specimen. The support distance was set to 40 mm and the tests were conducted at 25 °C. Each test was performed with a sample size of *n* = 5.

### 2.6. Implant Fixation and Bone Grafting Evaluation

Cell-type blocks of simulated bone manufactured from polyurethane (Sawbones, Vashon, WA, USA) were used in this work. The ability of the BC to provide a suitable degree of bone filling in low-density autologous bone was assessed using simulated specimens with densities of 7.5 PCF (L), 12.5 PCF (M) and 20 PCF (H), as shown in Figure S3.

The simulated bone specimens were 60 mm in width, 60 mm in depth and 40 mm in height. The model implants each comprised an SUS 304 stainless steel rod with a diameter of 16 mm, hard chrome plating and a surface roughness (Ra) of 0.12 μm. The relationship between the thickness of the BC application and fixation strength was investigated by drilling pilot holes into the simulated bone having diameters of 16.4, 16.8 or 17.2 mm, with a constant depth of 24 mm.

During fixation trials, the cement was first injected into the pilot hole in the simulated bone to a height of approximately 12 mm, followed by insertion of the implant. A packing ring and the associated retaining washer were then attached to the implant to prevent excessive leakage of the BC from the opening. The packing ring retaining force was approximately 2 kN while the implant insertion force was approximately 400 N. The fixation process was completed within 60 s from the start of cement injection. After fixation, the packing and retaining washer were removed. In some cases, the packing remained fixed to the upper surface of the simulated bone because a small amount of cement had flowed out from the opening. This was considered to have no effect on the test results and so the cement overflow was left as is (Figure S4a). Fixation strength was evaluated by clamping the simulated bone with a washer having an inner diameter of 46 mm and conducting a pull-out test of the implant using a bolt (Figure S4b). Based on observations of the fracture sites on each fixed body after the pull-out test that evaluated the cement penetration status, the degree of bone augmentation was determined.

### 2.7. Tibial Tray Fixation Strength Comparison

This study also compared the strength and fracture characteristics of actual tibial trays and simulated bones fixed using the BC combined with commercially available artificial bone. The tibial trays were fixed using cemented implants (Johnson & Johnson Attune RP artificial knee joint system, size 4) and cementless implants (Johnson & Johnson Attune cementless artificial knee joint system, size 4). The tibial trays used are shown in Figure S5.

Figure S6 provides diagrams of the simulated bone specimens used in this work. Prior to drilling, a gap was created by offsetting the implant by 0.8 mm and the artificial bone material was applied to this gap. It should be noted that the tibial tray used for cementless fixation is larger than autologous bone and is normally inserted with a slight interference fit. However, since the simulated bone lacked the flexibility of autologous bone, this technique could not be applied in the present work. Therefore, the same 0.8 mm gap was employed as had been used in trials with the cemented fixation units and the implant was fixed using the artificial bone material. In addition to the newly developed BC formulation, a widely used commercially available BC (referred to herein as the comparative BC) and a bone filler (referred to as the comparative bone filler) were also used as artificial bone materials. The fixation method employed when working with the tibial tray and the simulated bone involved first applying the artificial bone material (mixed according to the specified process) to the tibial tray, inserting the simulated bone, and immediately applying a compressive force of approximately 100 N for 300 s. The amount of artificial bone material applied was sufficient to fill the gaps completely and any excess material that overflowed after filling the gaps was removed before curing. Each fixed specimen was immersed in a phosphate-buffered solution for 96–168 h, during which time curing took place. Following this, the cured specimens were dried for at least 24 h at 23 ± 5 °C and 75% relative humidity before testing. Testing was performed by inserting an extraction punch from the back surface of the fixed body and pushing it out to simulate the removal of the implant from the simulated bone. This process was based on the method previously reported by Grupp et al. [38] and is illustrated in Figure S7.

### 2.8. Statistical Analysis

Welch’s *t*-test was used to compare the developed product with the reference product. The significance level was set at α = 0.05. Variables analyzed included compressive strength, compressive modulus, flexural strength, and flexural modulus obtained from both groups. In experiments using simulated bones of varying densities, failure modes and fixation strength were assessed qualitatively. For experiments in which tibial trays were fixed to simulated bones using either the developed or reference product, *t*-tests were performed stratified by fixation method and type of bone cement.

## 3. Results and Discussion

### 3.1. Preparation of Raw Materials for BC

**Figure 1:**
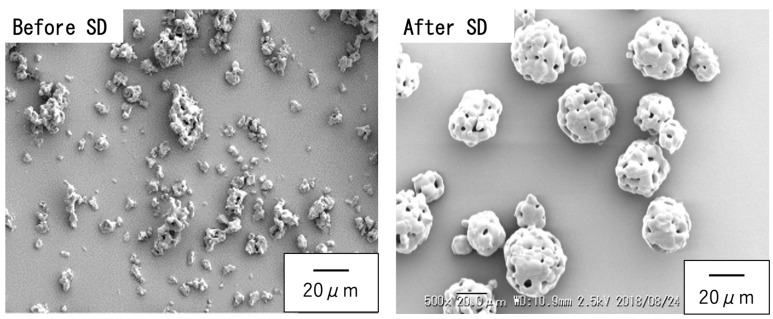
SEM images of β-TCP particles synthesized by solid-state reaction (**left**) before spray drying, showing irregular morphology and sizes of 2–5 µm, and (**right**) after spray drying and sintering, showing spherical porous granules and sizes of 15–30 µm.

Figure 1 shows SEM images of the β-TCP powder synthesized via a solid-state reaction and having the composition summarized in Table 1. These images show irregularly shaped particles with sizes ranging from 2 to 5 µm. After spray-drying and subsequent sintering, porous, spherical granules were obtained with sizes in the range of approximately 25–40 µm. These spherical particles exhibited significantly improved flowability and also showed better mixing efficiency and uniform reactivity when combined with the cyanoacrylate. The evident surface roughness of this material was also expected to enhance the adhesion between the particles and the cyanoacrylate.

**Figure 2:**
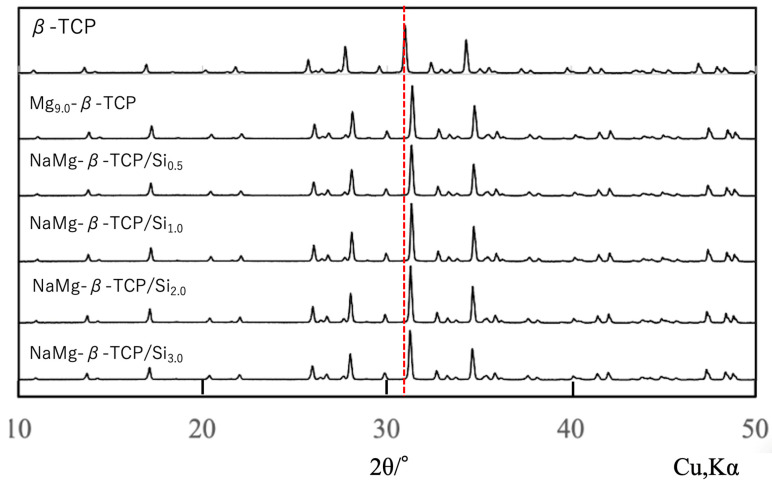
XRD patterns for spherical, porous, sintered β-TCP, Mg_9.0_-β-TCP and NaMg-β-TCP/Si particles. All samples produced characteristic β-TCP peaks without secondary phases. The red dashed line indicates the position of the main diffraction peak of β-TCP, showing that all diffraction peaks are shifted.

Figure 2 shows XRD patterns for porous, spherical, sintered β-TCP, Mg_9.0_-β-TCP and NaMg-β-TCP/Si particles. All samples generated characteristic diffraction peaks corresponding to a β-TCP structure (ICDD No. 55-0898), with no evidence of secondary HAp or β-Ca_2_P_2_O_7_ phases. From the figure, it can be seen that the X-ray diffraction peaks of the samples with various ions substituted into the solid solution are clearly shifted in position compared to the X-ray diffraction peaks of β-TCP. The peaks obtained from the Mg-substituted and Si-substituted samples were shifted to higher angles, suggesting that Si atoms were substituted at P(1) sites while Mg was substituted at Ca(5) sites and Na was incorporated into the Ca(4) vacancies for charge compensation. Through Rietveld analysis of the obtained X-ray diffraction patterns, we have revealed insights into the elucidation of these substitution sites. These structural changes were consistent with those observed in previous work by the authors [37].

To evaluate the possibility of Al_2_O_3_ contamination originating from abrasion of the Al_2_O_3_ milling pot during long-duration wet milling, the phase composition of the milled powders was re-examined. Special attention was paid to the characteristic diffraction peaks of corundum-type Al_2_O_3_ at 2θ = 25.6°, 35.1°, 43.3°, 52.5°, and 57.5°. The weak signals observed around 35° and 45° in the XRD patterns (Figure 2) overlapped with the intrinsic β-TCP reflections, such as (214), (300), (22-4), and (24-3), and no additional reflections attributable to Al_2_O_3_ were detected.

Furthermore, EPMA and SEM-EDS analyses revealed that the Al content in both the milled powders and the sintered granules was below the detection limit, confirming that alumina contamination, if present, was negligible. These results indicate that the observed diffraction signals arise solely from β-TCP and are not related to Al_2_O_3_ abrasion.

**Figure 3:**
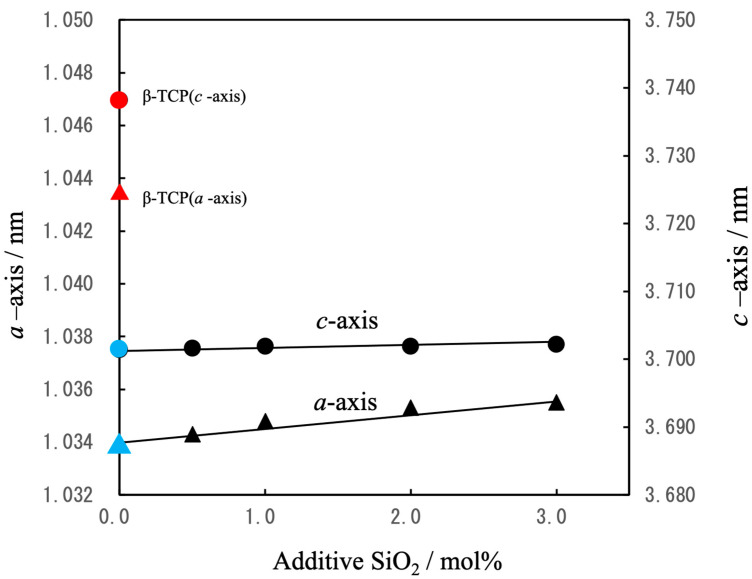
Lattice constants (*a*, *c*) for β-TCP(▲,●), Mg_9.0_-β-TCP (▲,●)and NaMg-β-TCP/Si (▲,●)as determined by XRD (△: *a*-axis, ○: *c*-axis). Mg substitution reduced both lattice parameters whereas Si and Na substitution slightly increased the *a*-axis parameter.

The lattice constants for these samples are plotted as functions of the amount of SiO_2_ in Figure 3. The lattice parameters for the pure β-TCP were found to be *a* = 1.0435 nm and *c* = 3.7372 nm, in good agreement with constants previously reported by the authors [39]. In the case of the Mg-substituted β-TCP (in which 9.09 mol% of the Ca atoms at Ca(5) sites were replaced with Mg), the constants were *a* = 1.0341 nm and *c* = 3.7034 nm, consistent with data obtained from other studies [40]. Upon the additional substitution of Si at the P(1) sites, with Na added for charge compensation at the Ca(4) vacancies, increasing the Si content to 3 mol% of the total P caused the *a*-axis constant to slightly increase while the *c*-axis constant remained essentially unchanged. The absence of secondary phases and the systematic changes in the lattice constants indicated that these samples consisted of solid solutions based on the β-TCP crystal structure [41].

**Figure 4:**
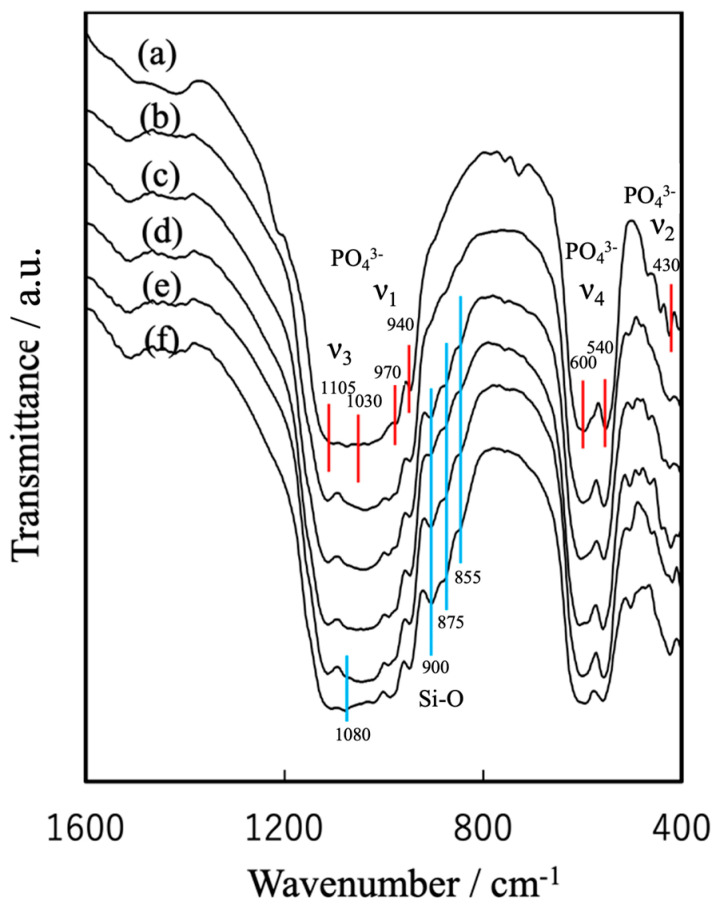
FT-IR spectra of spherical, porous, sintered particles of (a) β-TCP, (b) Mg_9.0_-β-TCP, (c) NaMg-β-TCP/Si_0.5_, (d) NaMg-β-TCP/Si_1.0_, (e) NaMg-β-TCP/Si_2.0_ and (f) NaMg-β-TCP/Si_3.0_. The absorption of the PO_4_ group is shown by the red line, and the absorption of Si-O is shown by the blue line.

Figure 4 provides FT-IR spectra of the various specimens. The β-TCP generated characteristic peaks related to the vibrations of phosphate groups at 430 cm^−1^ (ν_2_), 540 and 600 cm^−1^ (ν_4_), and 940 and 970 cm^−1^ (ν_1_), as well as at 1030 and 1105 cm^−1^ (ν_3_) [42]. A peak at 3570 cm^−1^ related to -OH absorption and attributable to hydroxyapatite was not observed, nor was a P–O–P peak assignable to β-Ca_2_P_2_O_7_ and normally found at 727 cm^−1^. These results confirm that the β-TCP was present as a single phase. The FT-IR spectrum of the Mg_9.0_-β-TCP was essentially identical to that of the β-TCP, indicating that the substitution of Ca ions by Mg ions at the Ca(5) sites did not alter the fundamental phosphate vibrational modes. In contrast, the NaMg-β-TCP/Si spectrum exhibited distinct features attributable to the incorporation of SiO_4_ units at PO_4_ sites. In addition to the characteristic phosphate absorption peaks, this sample produced bands corresponding to the asymmetric stretching vibration (ν_3_) of Si–O at approximately 1080 cm^−1^ as well as the symmetric stretching vibration (ν_1_) of Si–O at 855, 875 and 900 cm^−1^ [37].

The β-TCP spectrum shows characteristic phosphate peaks while the NaMg-β-TCP/Si spectra display peaks related to Si–O vibrations at 855, 875, 900 and 1080 cm^−1^.

**Figure 5:**
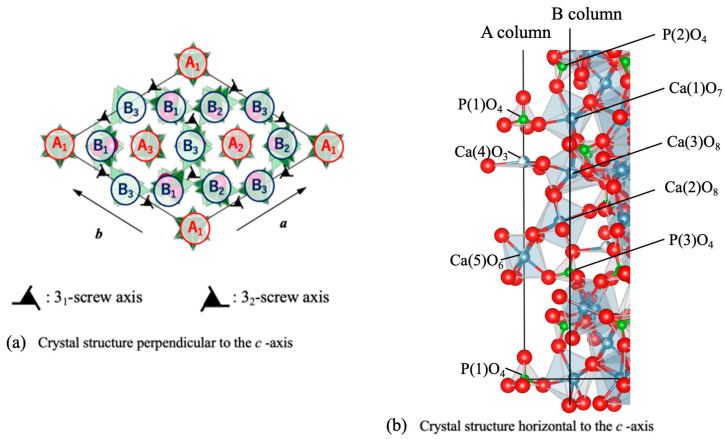
Schematic representation of β-TCP crystal structure. (**a**) Atomic arrangement along the *a*-axis, highlighting the A- and B-columns and (**b**) crystal structure with half the length relative to the *c*-axis direction, showing Mg ions substituted at Ca(5) sites, Na ions at Ca(4) sites and Si ions at P(1) sites [37,39,40,41]. A1..B1.. indicates A column and B column respectively, with the subscript numbers indicating the height position (**1**/3*c*, **2**/3*c*, **3**/3*c*) on the screw axis of *c*-axis. Each ion is represented by a sphere: Ca ions in gray, P ions in green, and O ions in red.

Figure 5 illustrates the β-TCP crystal structure. Figure 5a shows the atomic arrangement along the *a*-axis for a hexagonal setting. The β-TCP structure comprises linear A-columns (P(1)-Ca(4)-Ca(5)-P(1)-□(vacancy)-Ca(5)) with zigzag B-columns (P(2)-Ca(2)-Ca(1)-Ca(3)-P(3)-…) surrounding the A-columns to form tunnel-like structures [40]. In the Mg_9.0_-β-TCP, Mg^2+^ ions (having a radius of 0.065 nm and so smaller than Ca^2+^ ions with a radius of 0.099 nm) were substituted at Ca(5) sites in conjunction with octahedral coordination, leading to reductions in both the *a* and *c* lattice constants (see Figure 5b) [37]. In the Si-substituted samples, Si^4+^ ions (0.041 nm) partially replaced P^5+^ ions (0.034 nm) at P(1) sites, with Na^+^ ions (0.095 nm) compensating for the charge at the underlying Ca(4) vacancies. These substitutions caused a slight expansion in the *a*-axis while leaving the *c*-axis nearly constant. These mechanisms by which Mg, Na and Si were substituted into the β-TCP are consistent with those in previous reports [37,39,40].

### 3.2. BC Setting Time and Peak Temperature

**Figure 6:**
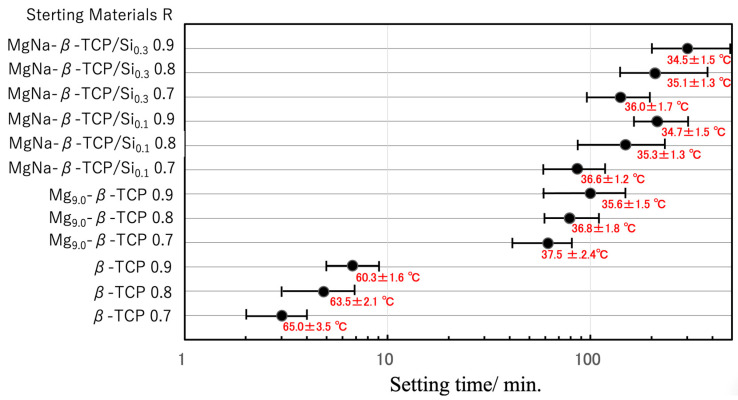
Setting times and peak exothermic temperatures of BC specimens prepared from β-TCP, Mg_9.0_-β-TCP or NaMg-β-TCP/Si particles and a cyanoacrylate at L/P ratios of 0.7–0.9 mL/g. The setting time axis is displayed on a log scale. The error bars indicate the short and long setting times, while ‘●’ indicates the average setting time. Peak exothermic temperatures are shown as mean ± standard deviation.

**Figure 7:**
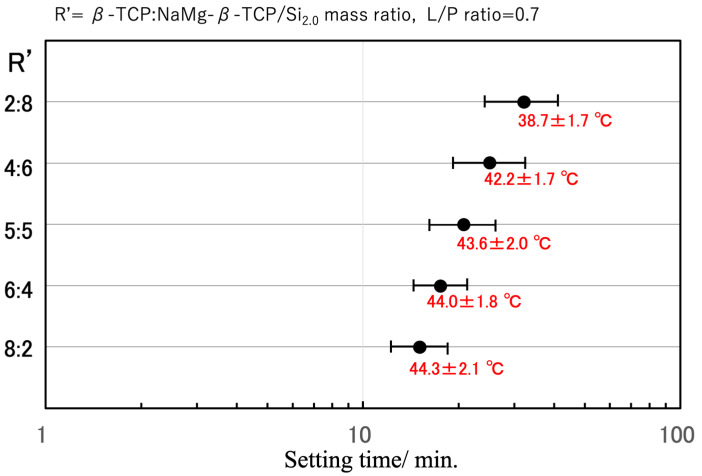
Optimization of setting behavior based on combining β-TCP and NaMg-β-TCP/Si_2.0_ in various ratios. The setting time axis is displayed on a log scale. The error bars indicate the short and long setting times, while ‘●’ indicates the average setting time. Peak exothermic temperatures are shown as mean ± standard deviation.

The setting time and peak exotherm temperature for the BC specimens prepared by mixing the aforementioned particles with the cyanoacrylate at L/P ratios of 0.7–0.9 mL/g are presented in Figure 6 and Figure 7.

Figure 6 summarizes the effects of the L/P ratio. In the case of the β-TCP-based particles, setting occurred within 3–7 min regardless of the cyanoacrylate content, with peak temperatures ranging from 60 to 65 °C. Cyanoacrylate generally polymerizes rapidly via an anionic mechanism. In this process, water molecules act as bases (OH^−^) that attack the electron-deficient double bond of the cyanoacrylate monomers, generating carbanions and propagating the chain polymerization [43,44]. To clarify the effect of the surface acidity of the particles on cyanoacrylate polymerization, BC mixtures incorporating Mg_9.0_-β-TCP and NaMg-β-TCP/Si were evaluated. When the Mg_9.0_-β-TCP was combined with the cyanoacrylate under identical conditions, the setting time was significantly increased, to approximately 60 min, while the peak temperature was decreased to an average of 38 °C. Increasing the cyanoacrylate proportion in the mixture further extended the setting time to approximately 100 min and reduced the exothermic peak temperature to approximately 36 °C. In the case of the NaMg-β-TCP/Si particles, the retardation effect was even more pronounced, with setting times extending from 85 to 120 min and peak temperatures in the range of 34–36 °C. These results confirm that the substitution of Mg, Na and Si into the β-TCP lattice effectively suppressed the exothermic polymerization of the cyanoacrylate, thereby extending the workable time while simultaneously reducing the reaction temperature [43,44]. Similar strategies have been reported with regard to other composite systems, such as magnesium phosphate cements combined with alginate hydrogels to regulate curing kinetics and improve clinical handling [45]. Figure S8 shows the relationship between the curing reaction time and exothermic temperature of cyanoacrylate mixtures with β-TCP, Mg-β-TCP, and Na/Mg-β-TCP as supplementary data.

With the aim of achieving an optimal setting time on the order of 20 min, the fast-curing β-TCP and slow-curing NaMg-β-TCP/Si_2.0_ were combined (see Figure 7). A 5:5 β-TCP:NaMg-β-TCP/Si_2.0_ mass ratio resulted in the desired setting time and maintained a relatively low peak temperature of 43.6 ± 2.0 °C. The setting time could also be tuned by changing the proportion of Si in the particles and varying the L/P ratio.

**Figure 8:**
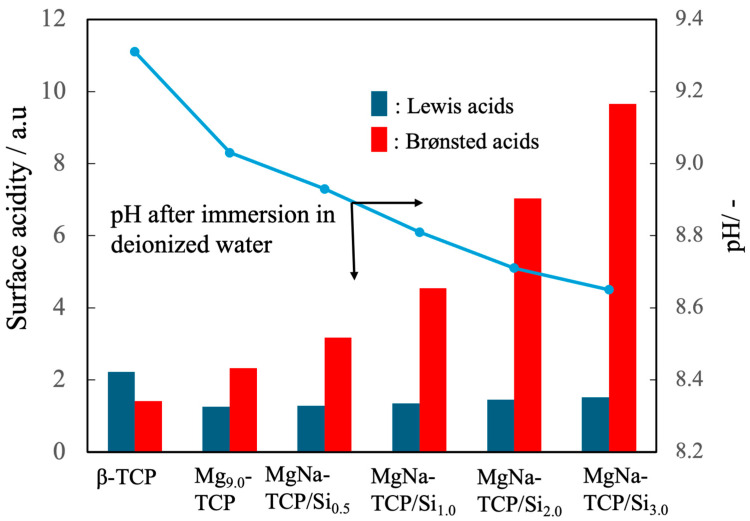
Surface acidities of β-TCP, Mg_9.0_-β-TCP and NaMg-β-TCP/Si particles as measured by pyridine adsorption, showing increases in the quantity of Brønsted acid sites with increasing Si substitution, and pH after immersion in deionized water. Note that the analysis of Brønsted acids/Lewis acids is semi-quantitative, so statistical processing is not possible. The arrows indicate the relationship between the horizontal axis and the vertical axis.

The surface acidities of the BC powders were further characterized by pyridine adsorption, with the results presented in Figure 8. The pure β-TCP was found to have negligible surface amounts of both Brønsted and Lewis acid sites. In contrast, the Mg_9.0_-β-TCP and NaMg-β-TCP/Si demonstrated marked increases in Brønsted acid site density that correlated with the extent of Si substitution. Moreover, immersion tests revealed a decrease in the pH of the supernatant following 10 min of stirring in deionized water. Taken together, these findings indicate that the suppression of cyanoacrylate polymerization that was observed can be ascribed to the presence of protons at Brønsted acid sites that effectively neutralized hydroxide ions derived from ambient moisture, thereby hindering the initiation of the anionic chain polymerization reaction [43,44].

### 3.3. BC Disintegration Resistance and Injectability

**Figure 9:**
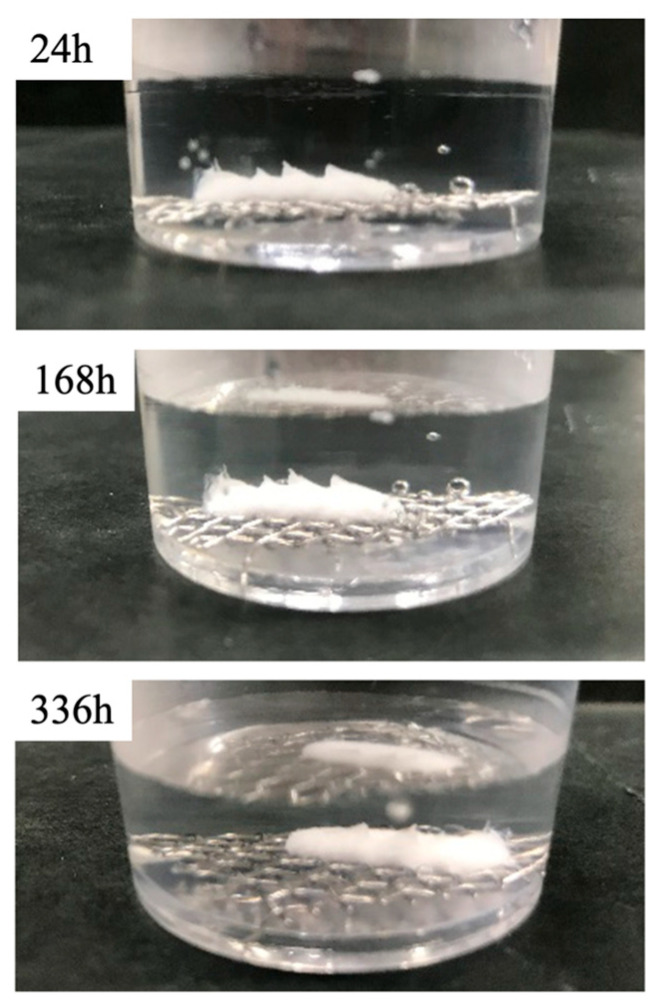
Disintegration resistance of BC paste incorporating β-TCP: NaMg-β-TCP/Si_0.5_ particle mixture with ratio of 6:4 in saline over a 336 h trial. The specimen exhibited a minimal mass loss on the order of 0.3%.

Figure 9 shows the resistance of the PC paste to disintegration in a saline solution. This prolonged immersion in physiological saline demonstrated that a paste comprising a β-TCP: NaMg-β-TCP/Si_0.5_ mixture with a 6:4 ratio and with an L/P ratio of 0.9 mL/g exhibited remarkably high resistance to disintegration, with a mass loss as low as 0.30 ± 0.12% after 336 h. This stability is of particular importance in clinical settings, where it is important to maintain cement integrity after the incorporation of the material in the body, so as to ensure mechanical support during the initial stages of bone regeneration.

**Figure 10:**
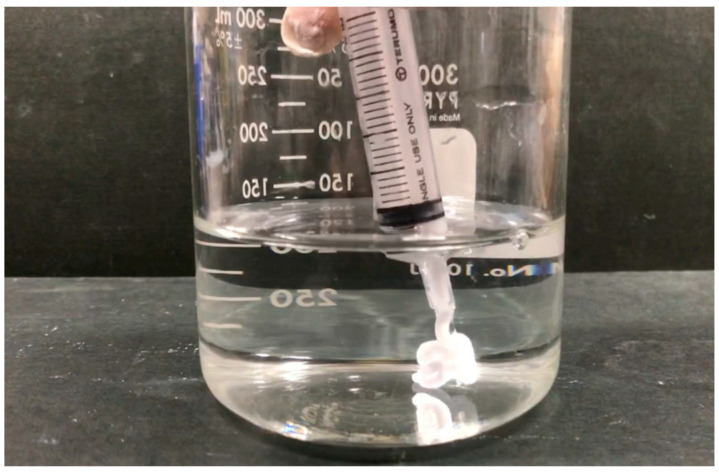
Injectability of BC paste demonstrated by extrusion through syringe into saline solution while maintaining structural integrity and forming superficial reaction layer.

In addition to stability, the BC paste exhibited excellent injectability and was found to readily extrude through a syringe while retaining its form upon contact with saline (see Figure 10). The immediate formation of a superficial reaction layer indicated an early physicochemical interaction with the surrounding environment that could act as a precursor for osteoconductive behavior and subsequent integration with host bone tissue. This ability to deliver the paste in a minimally invasive manner while maintaining structural stability would be advantageous in the case of clinical applications to irregular bone defects or confined surgical fields. Compared with conventional PMMA-based BC formulations that tend not to demonstrate resorption or osteoconductivity and generate high exotherm temperatures, the present BC showed superior chemical stability, smooth injectability and inherent bioactivity derived from the β-TCP. Furthermore, unlike brittle CPCs with poor injectability and insufficient mechanical strength, this composite combined reliable compressive performance with favorable handling. These dual advantages suggest that this material could function as a next-generation alternative for minimally invasive bone repair and augmentation.

### 3.4. Curing Behavior of the Composite

**Figure 11:**
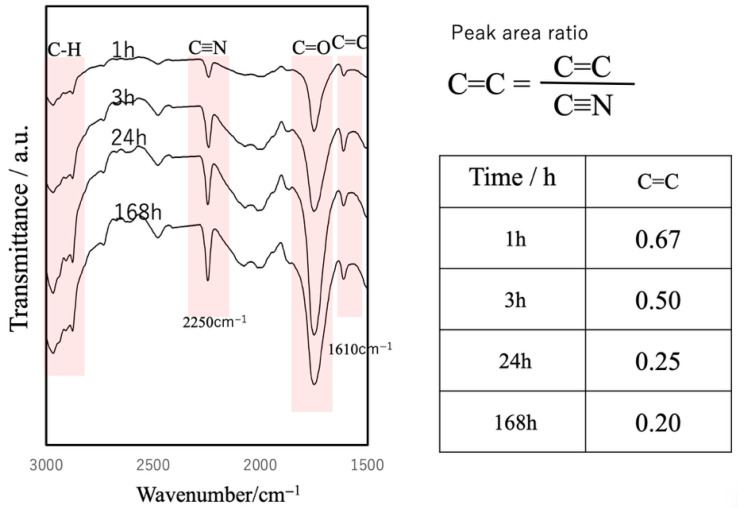
FT-IR spectra of cured BC cross-sections at different curing times, showing progressive cyanoacrylate polymerization.

FT-IR analysis of the internal cross-sections of cured BC specimens at different time points revealed an increase in peaks corresponding to C≡N, C=O and C–H and a decrease in C=C peaks, confirming the progressive polymerization of the cyanoacrylate (Figure 11) [43,44].

**Figure 12:**
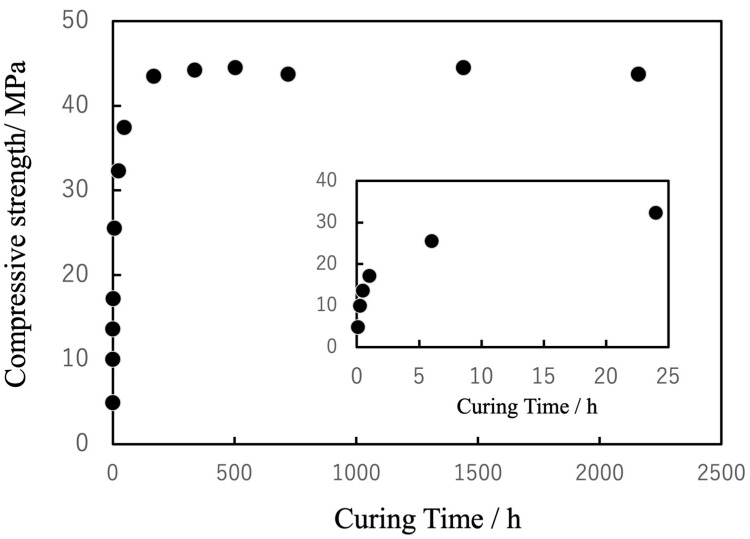
Changes in compressive strength of cured BC in PBS (pH 7.4) as function of curing time, stabilizing at approximately 40 MPa after 144 h.

The compressive strength of the specimens was found to increase along with this curing process (Figure 12). Specifically, the strength reached approximately 50% of the final value within 6 h, approximately 70% of this value after 24 h, and plateaued at approximately 40 MPa after 144 h. This final strength exceeded that of cancellous bone, indicating the ability of the material to provide reliable structural support. In a clinical setting, this gradual strength development would be advantageous as it would ensure sufficient workability during surgery while achieving stable fixation within the timeframe critical for initial bone healing. In contrast to the rapid but biologically inert curing of PMMA-based BC, the present system achieves a favorable balance of injectability, bioactivity and mechanical reliability.

**Figure 13:**
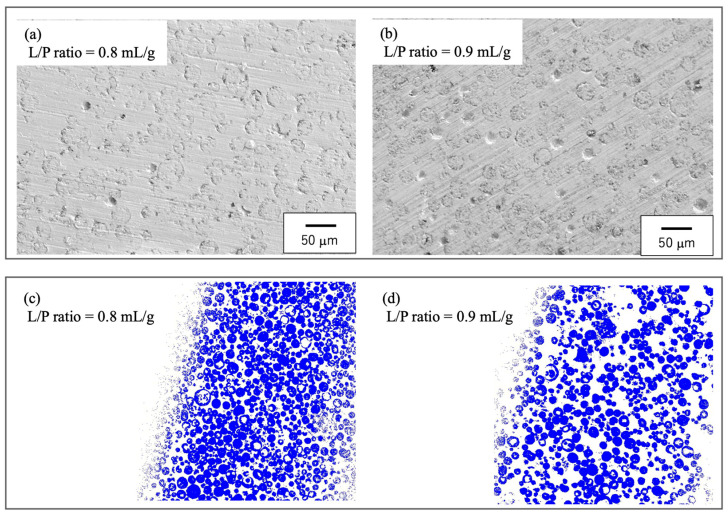
Microstructure of cured BC. (**a**,**b**) SEM images showing the dispersion of the spherical, porous particles and (**c**,**d**) EPMA elemental maps of Ca confirming uniform particle distributions. The white area represents the cyanoacrylate matrix, while the blue area indicates where calcium elements are distributed; (**a**) L/P ratio = 0.8 mL/g, (**b**) L/P ratio = 0.9 mL/g, (**c**) L/P ratio = 0.8 mL/g, (**d**) L/P ratio = 0.9 mL/g.

Figure 13 presents low- magnification SEM and EPMA images of the cured specimens. Figure S9 shows high-magnification SEM images of the obtained samples. These images confirm that the sintered particles were uniformly dispersed within the cyanoacrylate matrix. The apparent density of these samples, as determined by the Archimedes principle, was 1.65 ± 0.13 g/cm^3^, corresponding to a porosity of 43–45%. This porosity is primarily attributable to the presence of the sintered particles. This porous architecture would be expected to promote a ductile fracture response under compression as well as bone ingrowth. Nevertheless, concerns regarding possible inflammatory responses or cytotoxicity from cyanoacrylate degradation products remain. The surface of the sample immersed in PBS solution for an extended period showed dissolution of the exposed β-TCP particles, resulting in a concave depression in that area. Furthermore, the surface of the sample after the compression test revealed cracks and destruction of β-TCP particles following the application of stress to the sample (see Figure S9c,d). Although the present in vitro results demonstrate favorable stability and compatibility, further in vivo investigations will be essential to clarify the long-term degradation behavior and confirm clinical safety. Elemental mapping of Ca by EPMA further verified the spatial distribution of these particles, consistent with the SEM findings. It should be noted that, in the case of samples fabricated with a high L/P ratio, the sintered particles exhibited a tendency to aggregate more densely within the matrix.

### 3.5. Mechanical Properties of Cured BC

**Figure 14:**
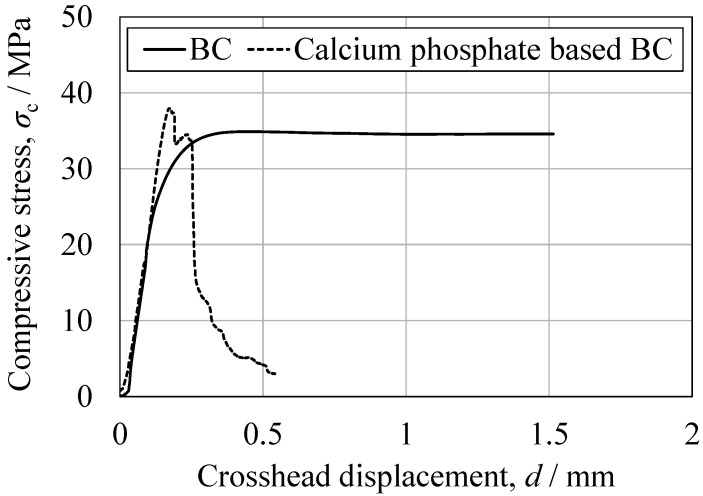
Representative compressive stress as function of crosshead displacement for the BC specimen and the CPC specimen.

**Figure 15:**
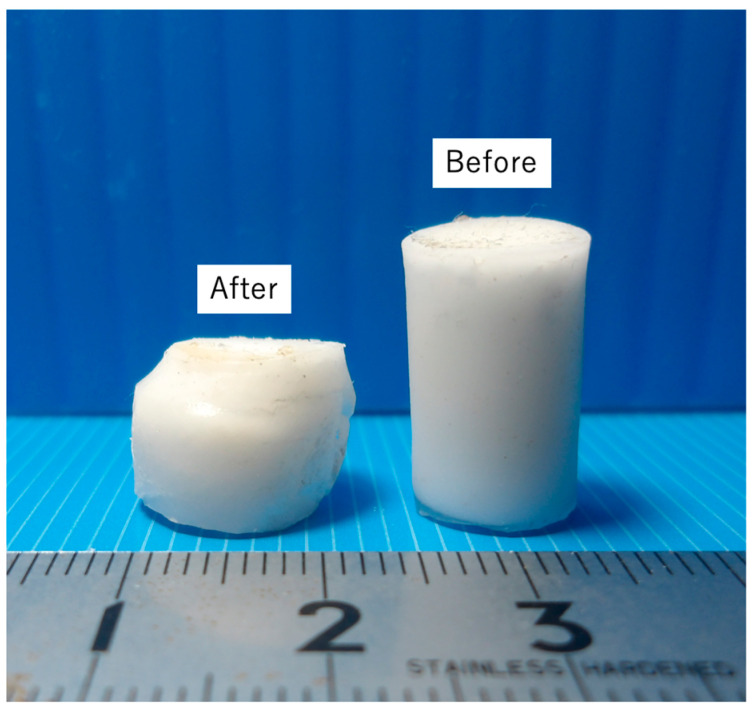
Appearance of BC specimens before and after compression, indicating ductile fracture.

**Figure 16:**
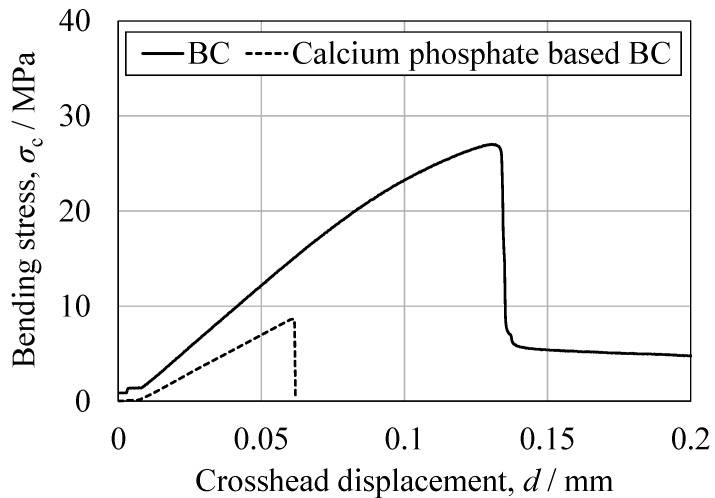
Representative bending stress as a function of crosshead displacement for the BC specimen and the CPC specimen.

**Figure 17:**
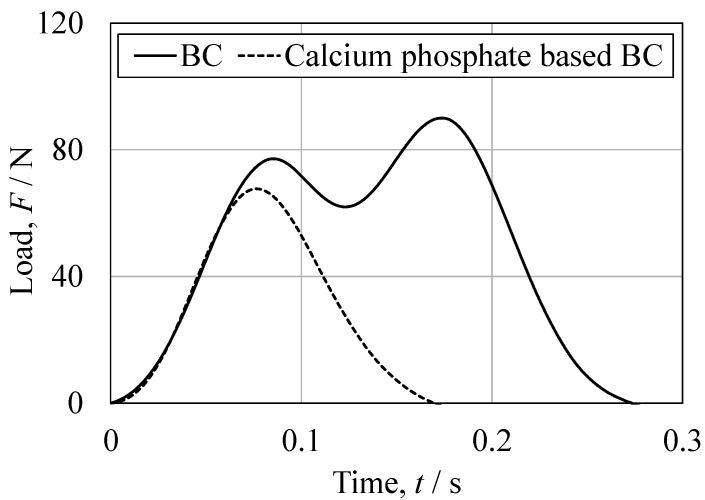
Representative load-time relationship obtained from the Charpy impact tests for the BC specimen and the CPC specimen.

**Figure 18:**
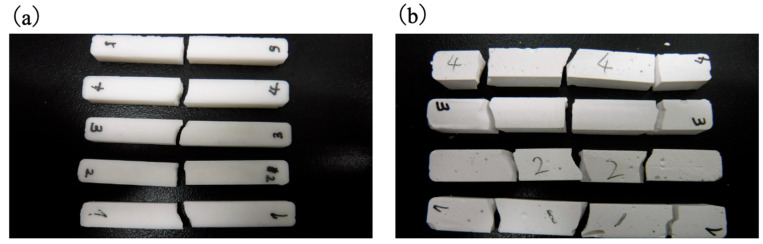
Images showing (**a**) BC specimens fractured into two pieces and (**b**) CPC specimens fractured into multiple fragments following impact.

The mechanical properties of the cured BC specimens were characterized by performing tensile, compression, three-point bending, torsion, fatigue and impact tests, with the stress–stroke diagrams and related photographs in Figure 14, Figure 15, Figure 16, Figure 17 and Figure 18. For comparison purposes, commercially available bone fillers were also examined by compression, bending and impact tests. The values determined for each of the mechanical properties are provided in Table 2. Examination of the tensile and compressive strengths reveals a marked asymmetry, in that the peak tensile stress was 10.2 MPa (*n* = 5) whereas the peak compressive stress reached 36.0 MPa, exceeding three times the tensile strength. This asymmetry is consistent with the general mechanical response of cementitious materials, in which stress concentration at internal pores under tensile loading promotes early crack initiation and brittle fracture. Despite exhibiting a compressive strength on the same order of magnitude as those of commercially available bone fillers, the BC was found to be marginally inferior and failed to satisfy the mechanical property requirements defined by ISO 5833 for PMMA-based bone cements [46]. In contrast to the brittle fracture observed under tensile loading, compression tests revealed ductile deformation, as evidenced by the gradual post-peak load decay. This behavior was substantially different from the abrupt stress drop seen in the case of a commercial bone filler comprising a calcium phosphate-based BC. This ductile response under compression suggests that the BC provides superior structural stability when subjected to compressive force (Figure 15). Comparable strategies to improve toughness have been demonstrated in work with hybrid CPC systems incorporating gelatin methacrylate and synthetic polymers [46]. Moreover, bioactive composites combining CPCs with bioglass or polymeric phases have shown enhanced mechanical reliability and biological performance [47]. These findings emphasize that the present β-TCP/cyanoacrylate system represents a distinct but complementary route to overcoming the inherent brittleness of conventional CPCs.

Based on the three-point bending tests, the peak bending strength of the present BC was approximately three times higher than that of a calcium phosphate-based BC. Bending involves the application of compressive stress on the upper surface of the specimen with a simultaneous tensile stress on the lower surface, with the neutral axis acting as the transition boundary. Hence, the relatively low tensile strength of the BC likely affected fracture initiation, a phenomenon that has also been observed in studies with other porous ceramic–polymer composites [24]. Direct tensile testing of the commercial filler could not be performed due to difficulties in specimen preparation. However, the bending and compression results for this material suggest even poorer tensile properties than those of the newly developed BC, consistent with the general behavior of calcium phosphate-based cements [47]. The curvatures of the stress–strain profiles near the peak load also differed significantly between materials. The BC exhibited a convex curve, suggesting limited plastic deformation prior to fracture, while the commercial filler showed a linear, elastic-brittle response up to failure. This observation is consistent with the greater deformation capacity of the BC in response to compression and aligns with earlier studies that assessed the effects of microstructural connectivity and pore morphology on composite fracture behavior [47].

The torsion test produced a peak surface shear stress of 17.5 MPa that was comparable to the shear strengths reported for adult cancellous bone (2–5 MPa) [47,48]. While this finding indicates that the BC approached the shear resistance of natural bone, further assessments based on a broader dataset will be required before drawing definitive conclusions. Shear strength is a critical parameter related to maintaining axial loads and preventing loosening during medical screw fixation. Although the present study does not specifically address the fixation characteristics of screws, the potential integration of medical screws with the BC assessed in this research represents a promising avenue for future research and clinical applications. Fatigue testing established that the BC could withstand repeated tensile–compressive cycling at ±3 MPa for 5 × 10^6^ cycles. This relatively low endurance limit under cyclic loading suggests that this material would be unsuitable for applications with sustained tensile fatigue stresses. However, in clinical uses where tensile loading is minimal, such as in tibial components of knee arthroplasty, this limitation may not be a concern. Robo et al. [49] reported that even a relatively low fatigue limit of approximately 3 to 5 MPa in low-modulus bone cement exceeds the physiological stress experienced by the spine during daily activities, suggesting the potential for clinical applications. Based on this finding, the present BC may similarly be considered a promising candidate for use as a low-stress artificial bone material in clinical settings.

Impact testing provided further insights into the fracture mechanics of this material. The load–time profile obtained from the BC showed two distinct load peaks, with the delay between hammer contact and the second peak being longer than that of the commercial filler (Figure 17). It appears that crack propagation after the initial fracture was slower in the BC and that the energy absorption capacity of the material was greater. These findings are consistent with the hypothesis that ductility under compression contributes to impact resistance [50]. The fracture patterns also differed markedly between the materials. (Figure 18) The BC typically fractured into two pieces with cracks initiating at both the hammer and fulcrum points, whereas the commercial filler fractured into four pieces with cracks predominantly originating from the fulcrum.

Taken together, these results show that the BC exhibited higher ductility and an enhanced energy absorption capacity under compression compared with the conventional bone filler. Even so, the tensile and fatigue properties of the former would limit its use in certain applications. This combination of properties may make the present BC formulation a promising candidate for load-bearing applications dominated by compressive stress, provided that the tensile limitations are accounted for in design and surgical planning.

### 3.6. Pull-Out Strengths of Implant–Bone Complexes Fabricated Using Simulated Bone Blocks

The pull-out strength for implant–bone complexes (hereafter referred to as “complexes”) fabricated using simulated bone blocks having a density of 20 PCF (H) was 4.70, 5.27 and 4.23 kN, in order of increasing pilot hole diameters. The complexes with a density of 12.5 PCF (M) had values of 4.26, 4.25 and 4.53 kN. In the case of the 7.5 PCF (L) simulated bone specimens, the large pore volume limited the strength that could be obtained when the same amount of BC was applied as had been used with the higher density bone samples. This occurred due to insufficient infiltration and a lower contact area. The maximum pull-out strength in such cases after optimizing increases in the BC mass was 1.59 kN. Notably, increasing the pilot hole diameter—equivalent to increasing the applied BC thickness—did not result in a significant change in strength. From this finding, it is evident that a thicker BC layer could potentially be used to fill irregular defects without compromising fixation performance.

**Figure 19:**
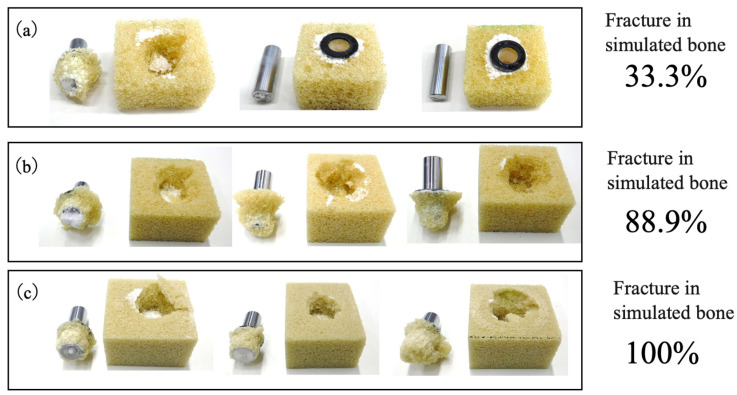
Representative fracture modes observed in three specimens selected from total of nine tests conducted for each density group. The fracture modes were determined through visual inspection. Images are shown for (**a**) L specimen with density of 7.5 PCF (0.12 g/cm^3^), (**b**) M specimen with density of 12.5 PCF (0.20 g/cm^3^) and (**c**) H specimen with density of 20 PCF (0.32 g/cm^3^).

The appearance of representative post-test specimens is shown in Figure 19. Nine datasets were acquired for each density level, revealing distinct fracture patterns. In trials using the low-density simulated bone, failure frequently occurred at the BC–implant interface whereas, in the case of the higher-density bone, fractures tended to occur either within the bone structure itself or at the interface between BC-infiltrated and non-infiltrated regions. Nevertheless, no substantial difference in peak strength was observed among the samples exhibiting different failure modes, suggesting that the mechanical strength at the implant–bone cement interface was comparable to that of the simulated bone material.

**Figure 20:**
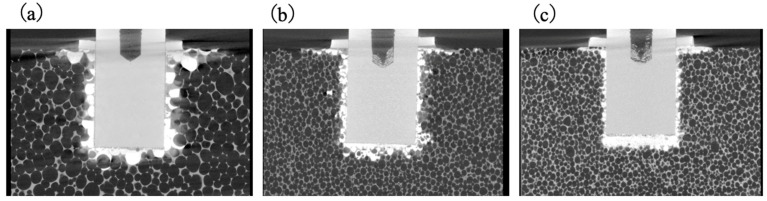
X-ray CT images showing BC penetration into porous simulated bones having (**a**) L, (**b**) M and (**c**) H density levels.

The causes of the strength variations were examined by preparing simulated bone- models based on replacing the metallic implant rods with PTFE rods of identical dimensions. This modification enabled X-ray computed tomography (CT) observations of the penetration of the BC into the porous structures. The CT images in Figure 20 demonstrate that the BC in the 7.5 PCF bone exhibited greater outflow through the large pores yet still filled the pores. Nevertheless, regions were observed at the implant–bone interface where the extent of BC infiltration was excessive, resulting in areas of reduced BC–implant contact. This finding suggests that the reduction in fixation strength can be attributed not to the fracture pattern itself but rather to diminished interfacial adhesion between the BC and the cylinder. This lower adhesion, in turn, resulted from capillary-driven BC outflow into the surrounding porous matrix.

**Figure 21:**
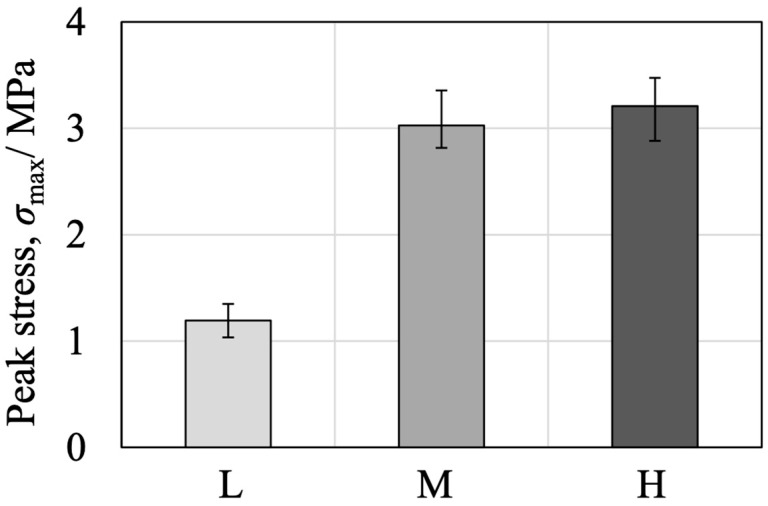
Tensile strength results of simulated bone blocks with different densities (L, M, H).

Because the low-density specimens showed fewer instances of internal fracture while some failures occurred within the simulated bone itself, the intrinsic strength of the bone analogue was also examined. Simulated bone blocks were cut into cross-sections having dimensions of 25 × 25 mm with a height of 40 mm, after which steel flanges were bonded to each end using an epoxy adhesive. The tensile strength of these specimens was then ascertained by pulling the flanges in opposite directions. As shown in Figure S10, internal fractures occurred in all density types. The tensile stress calculated from the peak load divided by the external cross-sectional area (neglecting porosity) for each sample type is provided in Figure 21. The 7.5 PCF (L) bone exhibited a markedly lower tensile strength compared with the 12.5 (M) and 20 PCF (H) samples.

These findings suggest that the markedly lower fixation strength observed in trials using the 7.5 PCF simulated bone during the pull-out test of the rod-shaped implant model corresponds to a similar trend in the intrinsic strength of the simulated bone itself. This implies that the fixation site in 7.5 PCF simulated bone was not in a critically weakened condition compared with those in the higher-density specimens. Conversely, considering that approximately one-third of the failures in the pull-out test of the rod-shaped implant model occurred within the simulated bone itself, it can be inferred that the fixation strength was comparable to the intrinsic strength of the bone. From this equivalence, it appears that the fixation site was not structurally deficient. Hence, this technology could be applied in clinical scenarios involving low-density autologous bone, such as in the treatment of osteoporotic conditions. Although some cases of BC–implant interfacial debonding were observed in the 7.5 and 12.5 PCF specimens, it is anticipated that adjusting the applied volume of BC such that the capillary-driven outflow capacity is exceeded could improve interfacial bonding. Such findings agree with previous studies reporting that an optimal adhesive volume and suitable pore-filling behavior are critical with regard to achieving stable implant fixation in low-density cancellous bone [51]. A two-step application process could be the best approach to regulating capillary forces at the interface. In this process, the low viscosity of the BC could be initially used to facilitate effective pore filling, followed by a secondary application that leverages the adhesive properties of the BC to achieve secure implant fixation. This approach could potentially provide fixation strengths exceeding those observed in the present study, and thus represents a promising avenue for future research.

### 3.7. Comparison of Fixation Strength in Tibial Tray–Simulated Bone Complexes Using Various Bone Substitute Materials

The fixation of implants was evaluated in both cemented and cementless tibial tray–simulated bone complexes, as shown in Figure S11, and the respective fixation strengths are summarized in Table 3. In the cemented configuration, fixation with the newly developed BC resulted in two distinct fracture patterns: complete fracture within the simulated bone or a combination of internal fracture with debonding at the BC–tibial tray interface. In contrast, fixation with the commercially available PMMA-based BC primarily resulted in internal fracture within the simulated bone, with minimal interfacial debonding. As shown in Table 3, the mean peak load for the PMMA-based BC was higher than that for the BC, in agreement with the observed fracture modes. The fixation strength obtained from the BC was approximately 71% of that achieved with the PMMA-based BC. This difference can be attributed to the superior cohesive and adhesive properties of the latter material in cemented arthroplasty applications [23,26].

Similar trends were observed in trials using the cementless configuration. As shown in Figure S12, fixation with the BC produced both internal fracture within the simulated bone and interfacial debonding, either between the BC and the implant or between the BC and the simulated bone. In the case of the commercially available bone substitute, internal fracture within the simulated bone was limited to the central boss of the tibial tray and the small region encircled by the four smaller bosses. Outside this region, all failures occurred at the fixation interface or within the bone substitute material itself. Notably, a comparison of the peak loads established that the BC provided approximately twice the fixation strength of the commercially available bone substitute (see Table 3). In addition to mechanical fixation, bioactive degradable composite cements integrating calcium silicate or phosphate phases have been reported to promote both osteoconductivity and gradual resorption [52]. Similarly, functional composites embedding bioactive glass have demonstrated tunable antibiotic release profiles while maintaining mechanical integrity [53]. Such approaches highlight potential avenues for expanding the functionality of β-TCP/cyanoacrylate systems in the future.

These results indicate that, while the BC provided lower fixation strength than the PMMA in cemented applications, its performance in the cementless configuration surpassed that of conventional bone substitute materials. Based on its initial fixation strength, this BC could have applications as a novel bone substitute in cementless total knee arthroplasty. By intentionally enlarging the pilot hole in a synthetic bone model designed for cementless fixation to simulate a segmental bone defect, and subsequently filling the void with BC to secure the tibial tray, this study demonstrates the potential clinical applicability of the BC as a defect-filling material for orthopedic implant fixation. The combination of adequate interfacial adhesion and structural support provided by this newly developed formulation suggests potential clinical benefits, particularly in scenarios where cementless fixation is desired to avoid cement-related complications [54,55,56,57].

Finaly, the present BC is a novel material exhibiting intermediate characteristics between PMMA-based BC and CPC. This study does not determine the feasibility of its final clinical use inside bone. Biological safety must be thoroughly verified before any clinical application is considered. Therefore, the current work represents an initial materials development study rather than a proposal for immediate clinical use.

## 4. Conclusions

This study developed a novel injectable composite bone cement composed of spherical, porous β-TCP granules and a cyanoacrylate adhesive. Through Mg, Na, and Si substitution, the surface acidity of β-TCP was effectively modulated, enabling controlled cyanoacrylate polymerization with an extended setting time and markedly reduced exothermic temperature. These improvements directly address the major limitations of conventional cyanoacrylate systems, namely excessively rapid curing and high thermal output.

The composite exhibited excellent injectability, high chemical stability, and compressive strength exceeding that of cancellous bone, together with ductile-like deformation, enhanced energy absorption, and notable impact resistance. These mechanical characteristics clearly surpass those of brittle CPCs and provide distinct advantages over PMMA-based cements, which lack bioactivity, are non-resorbable, and pose thermal risks during polymerization. However, the tensile and fatigue properties remain insufficient for high-load-bearing orthopedic applications, and the biological effects of cyanoacrylate degradation products warrant further investigation.

Future studies should focus on long-term in vivo evaluations to clarify degradation behavior, bone substitution capability, and overall biological safety and on refining the formulation to further improve tensile and fatigue resistance. Although this composite exhibits functional characteristics intermediate between PMMA bone cements and CPCs, the present work represents a preclinical materials development study. Additional biological validation will be essential before considering clinical translation.

**Key findings of this study**

1. Successful fabrication of an injectable β-TCP/cyanoacrylate composite bone cement.
2. Ion substitution (Mg, Na, Si) enabled controlled cyanoacrylate polymerization, extending setting time and reducing exothermic heat.
3. Excellent injectability and chemical stability suitable for minimally invasive procedures.
4. Compressive strength exceeding cancellous bone, with enhanced toughness-related properties such as ductility, energy absorption, and impact resistance.
5. Clear advantages over PMMA-based cements (lack of bioactivity, thermal risks) and CPCs (brittleness, limited injectability).
6. Remaining limitations include insufficient tensile/fatigue performance and the need to clarify potential inflammatory responses associated with adhesive degradation.

## Figures and Tables

**Table 1:** Chemical compositions of raw powder mixtures.

Sample	Blending Ratio							Chemical Formula
	CaCO_3_ /mol	(NH_4_)_2_HPO_4_ /mol	NaNO_3_ /mol	MgO /mol	SiO_2_ /mol	Vacancy:□ /mol	(Ca + Na + Mg + □)/(P + S) Molar Ratio	
Β-TCP	3.0000	2.0000	-	-	-	- (0.1429)	1.50 (1.571)	β-Ca_3_(PO_4_)_2_ (β-Ca_21.0_□_1.0_(PO_4_)_14_)
Mg_9.0_-β-TCP	2.7143	2.000	-	0.2857	-	0.1429	1.571	Ca_19.0_Mg_2.0_□_1.0_(PO_4_)_14.0_
NaMg-β-TCP/Si_0.5_	2.7143	1.9900	0.010	0.2857	0.010	0.1419	1.571	Ca_19.0_Mg_2.0_Na_0.07_□_0.93_(PO_4_)_13.93_(SiO_4_)_0.07_
NaMg-β-TCP/Si_1.0_	2.7143	1.9800	0.020	0.2857	0.020	0.1409	1.571	Ca_19.0_Mg_2.0_Na_0.14_□_0.86_(PO_4_)_13.86_(SiO_4_)_0.14_
NaMg-β-TCP/Si_2.0_	2.7143	1.9600	0.040	0.2857	0.040	0.1389	1.571	Ca_19.0_Mg_2.0_Na_0.28_□_0.72_(PO_4_)_13.72_(SiO_4_)_0.28_
NaMg-β-TCP/Si_3.0_	2.7143	1.9400	0.060	0.2857	0.060	0.1369	1.571	Ca_19.0_Mg_2.0_Na_0.42_□_0.68_(PO_4_)_13.58_(SiO_4_)_0.42_

**Table 2:** Summary of mechanical property values obtained from tensile, compressive, bending, torsional, fatigue testing of BC specimens and comparison data for commercial bone fillers.

Test Method	Mechanical Properties	Results (Mean ± SD)		*p*-Value (Welch’s *t*-Test) Significance Level *α* = 0.05
		BC	Calcium Phosphate Based BC	
Tensile	Peak tensile stress (MPa)	10.2 ± 1.2	―	―
	Elastic modulus (MPa)	4787 ± 590	―	―
Compression	Peak compressive stress (MPa)	36.0 ± 1.4	35.4 ± 9.6	0.893
	Elastic modulus (MPa)	3448 ± 390	4590 ± 459	0.017
Bending	Peak bending stress (MPa)	29.3 ± 8.5	9.29 ± 3.1	0.004
	Elastic modulus (MPa)	2169 ± 287	1353 ± 413	0.008
Torsion	Peak shear stress (MPa)	17.5 ± 3.3	―	―
Fatigue	fatigue life at 5 million cycles (MPa)	3.00	―	―

**Table 3:** Fixation strength of tibial trays secured to simulated bone using various bone cements.

Type of Fixation	Fixation Load (Mean ± SD)			*p*-Value (Welch’s *t*-Test) Significance Level *α* = 0.05
	BC	PMMA-Based BC	Calcium Phosphate Based BC	
Cemented fixation	4.57 ± 0.92	6.42 ± 0.57	―	0.006
Cementless fixation	6.14 ± 0.43	―	2.28 ± 0.24	<0.001

## Data Availability

The original contributions presented in the study are included in the article, further inquiries can be directed to the corresponding author.

## Supplementary Materials

The following supporting information can be downloaded at https://www.mdpi.com/article/10.3390/jfb16120458/s1, Figure S1: Schematic diagram of spray-drying equipment used to prepare spherical, porous granules; Figure S2: Dimensions and shapes of test specimens used in tensile, compressive, bending, torsion, fatigue and impact tests; Figure S3: Simulated bone blocks having densities of 7.5, 12.5 and 20 PCF with pilot holes; Figure S4: Schematics of fixture made from polyurethane foam to hold implant, showing (a) external view and (b) cross-sectional view including the cement filling; Figure S5: Photographic images of the (a) cemented and (b) cementless tibial trays used in this work; Figure S6: Schematic diagrams of (a) cemented and (b) cementless simulated bone specimens with pilot holes; Figure S7: Schematic of apparatus used for push-out test method as means of measuring fixation strength of tibial tray–bone complexes; Figure S8: Temperature-time curves for the thermal polymerization reaction of mixtures of β-TCP, Mg_9.0_-β-TCP, and NaMg-β-TCP/Si_0.3_; Figure S9: High-magnification SEM images of the samples; (a) The cyanoacrylate matrix; (b) β-TCP granular boundaries; (c) Surface of BC after 2160 h in PBS; (d) Surface of BC after compression testing; Figure S10:Photographic images of simulated bone specimens having (a) L, (b) M and (c) H densities after fracture during tensile tests; Figure S11: Photographic images of cemented tibial trays fixed with present BC in simulated bone. Images show specimens (a) prior to and (b–f) after push-out tests (samples 1–5); Figure S12: Photographic images of cementless type tibial trays fixed to simulated bone using the present BC. Images showing (a) specimen before the push-out test and (b) to (f) five samples after push-out test.
