# Supplementary material for: Evaluation of Composites Comprising Spherical, Porous, Sintered β-Tricalcium Phosphate Particles and Cyanoacrylate as Bone Cement
Source: J Funct Biomater. 2025 Dec 9;16(12):458. doi: 10.3390/jfb16120458 (PMC12733878; doi:10.3390/jfb16120458)
Supplement: Supplementary file 1 [file jfb-16-00458-s001.zip › jfb-3976432-supplementary.pdf]

Supplementary Information:

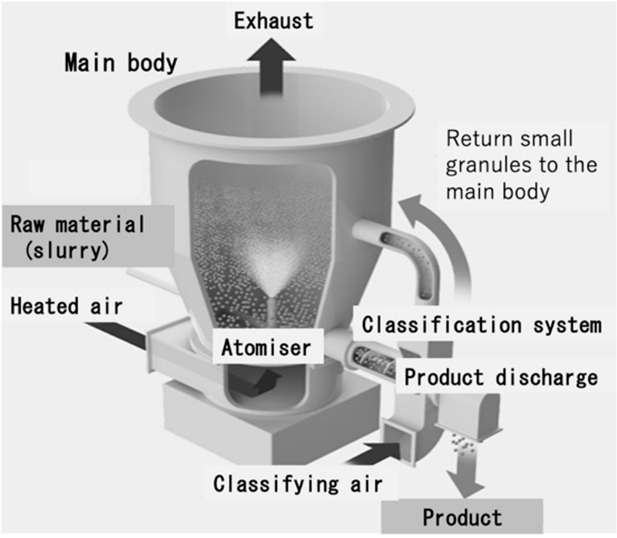

Figure S1. Schematic diagram of spray-drying equipment used to prepare spherical, porous granules.

Type of specimen	Plan view	Side view
Tensile	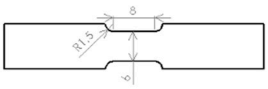	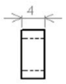
Compression	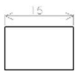	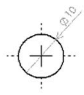
Bending	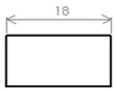	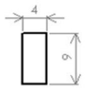
Torsion	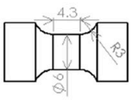	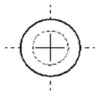
Fatigue	Same as tensile specimen	Same as tensile specimen
Impact	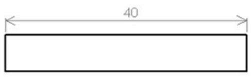	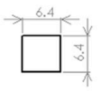

Figure S2. Dimensions and shapes of test specimens used in tensile, compressive, bending, torsion, fatigue and impact tests.

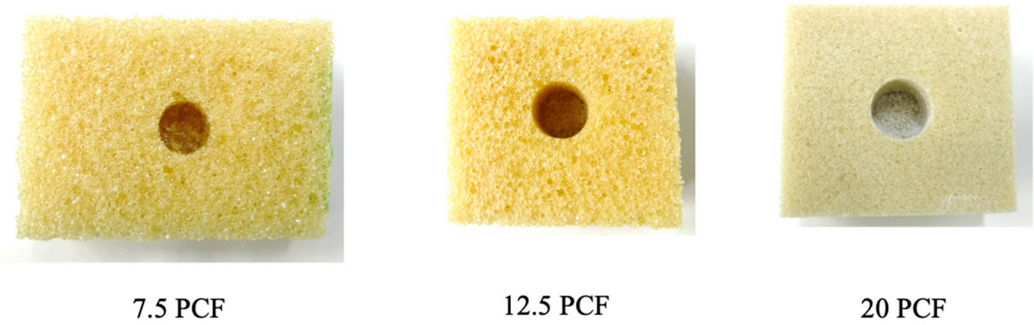

Figure S3. Simulated bone blocks having densities of 7.5, 12.5 and 20 PCF with pilot holes.

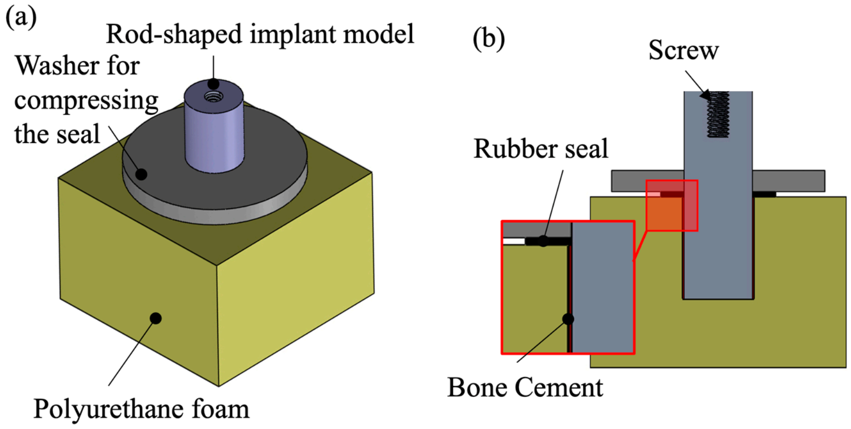

Figure S4. Schematics of fixture made from polyurethane foam to hold implant, showing (a) external view and (b) cross-sectional view including the cement filling.

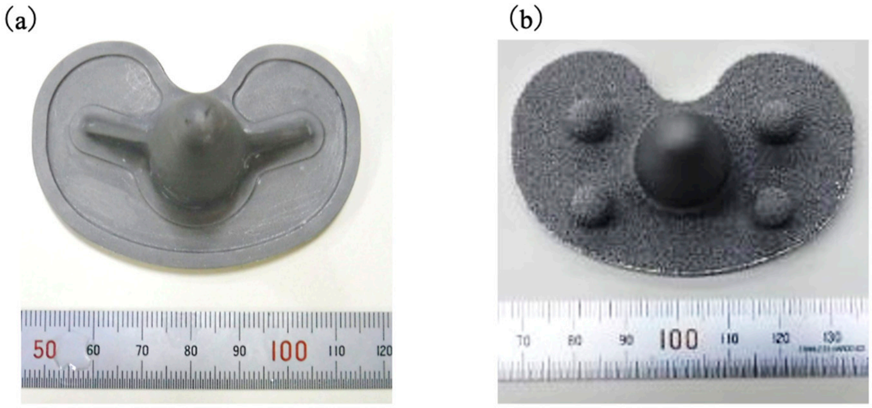

Figure S5. Photographic images of the (a) cemented and (b) cementless tibial trays used in this work.

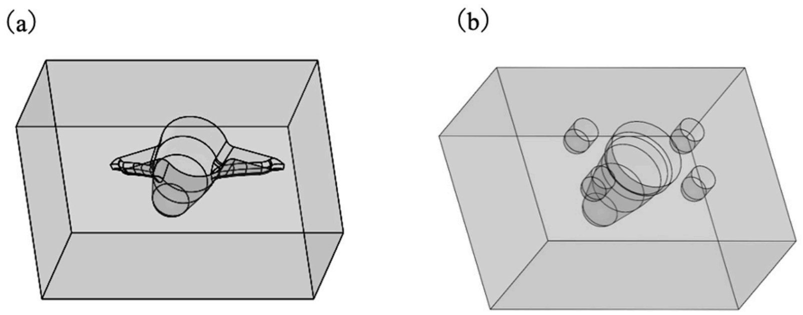

Figure S6. Schematic diagrams of (a) cemented and (b) cementless simulated bone specimens with pilot holes.

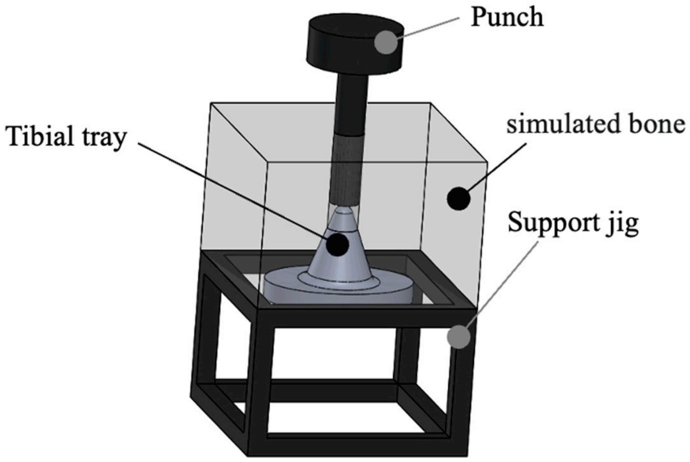

Figure S7. Schematic of apparatus used for push-out test method as means of measuring fixation strength of tibial tray–bone complexes.

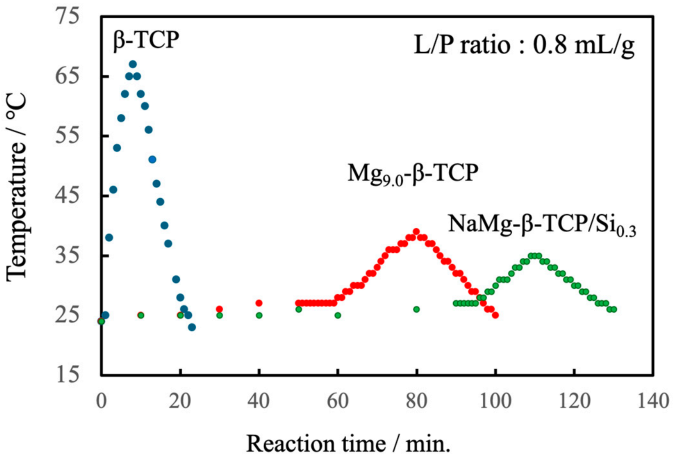

Figure S8 Temperature-time curves for the thermal polymerization reaction of mixtures of β-TCP, $Mg_{9.0}$-β-TCP, and NaMg-β-TCP/$Si_{0.3}$.

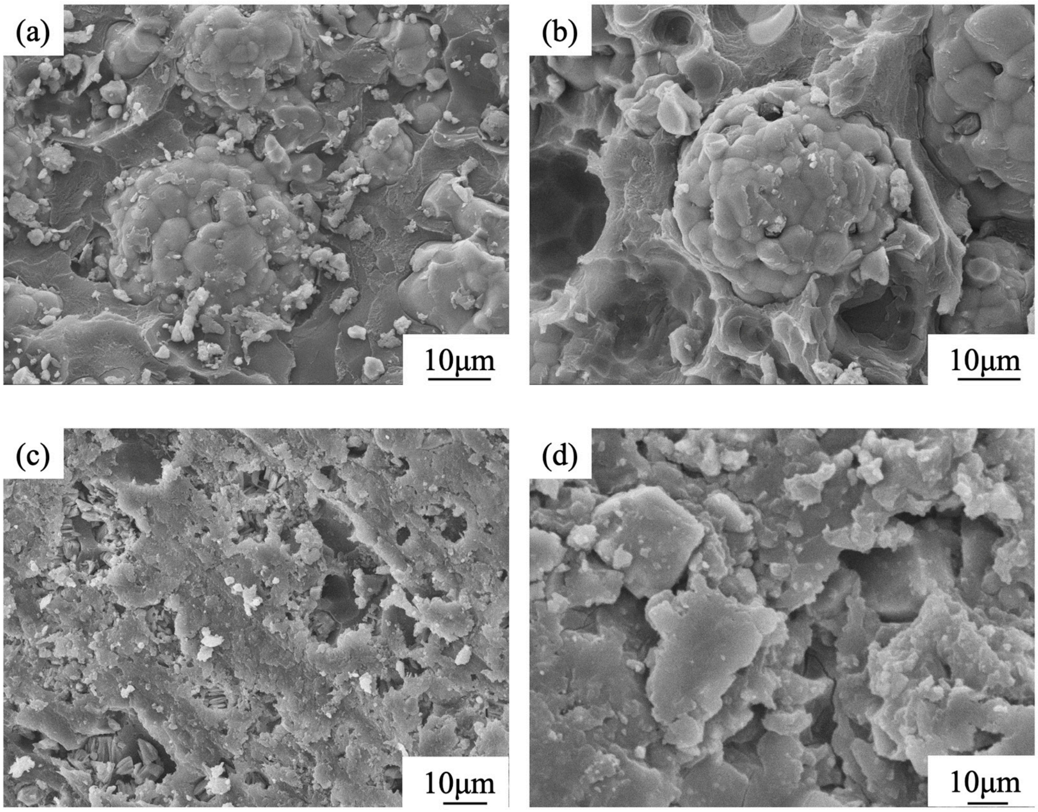

Figure S9 High-magnification SEM images of the samples; (a) The cyanoacrylate matrix; (b) β-TCP granular boundaries; (c) Surface of BC after 2160 h in PBS; (d) Surface of BC after compression testing.

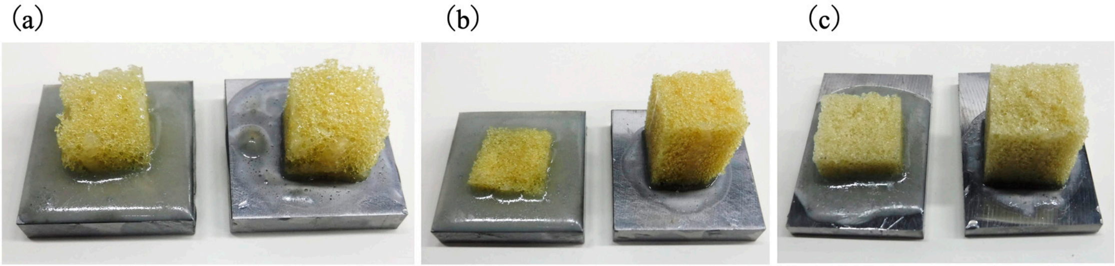

Figure S10. Photographic images of simulated bone specimens having (a) L, (b) M and (c) H densities after fracture in tensile tests.

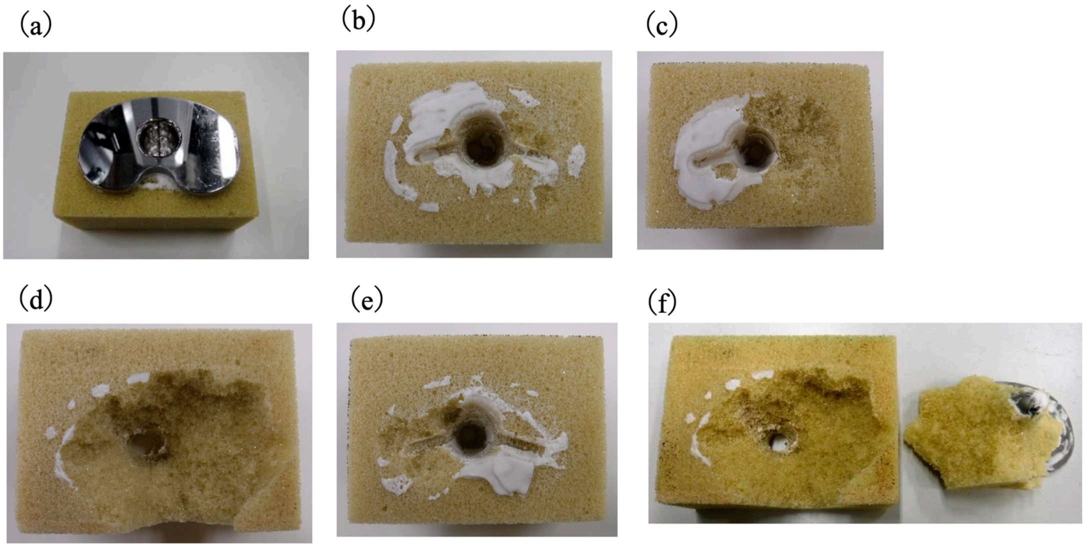

Figure S11. Photographic images of cemented tibial trays fixed with present BC in simulated bone. Images show specimens (a) prior to and (b–f) after push-out tests (samples 1–5).

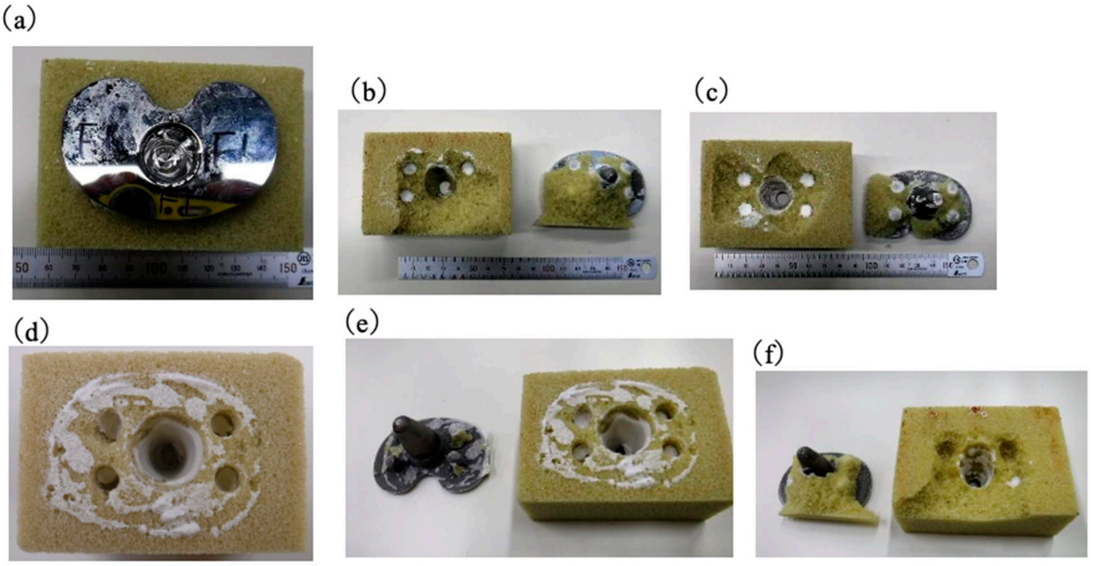

Figure S12. Photographic images of cementless type tibial trays fixed to simulated bone using the present BC. Images showing (a) specimen before the push-out test and (b) to (f) five samples after push-out test.
